# Metformin Synergistically Enhances Cisplatin-Induced Cytotoxicity in Esophageal Squamous Cancer Cells under Glucose-Deprivation Conditions

**DOI:** 10.1155/2016/8678634

**Published:** 2016-01-24

**Authors:** Hongliang Yu, Xiuhua Bian, Dayong Gu, Xia He

**Affiliations:** Department of Radiation Oncology, Jiangsu Cancer Hospital and Jiangsu Institute of Cancer Research, The Affiliated Cancer Hospital of Nanjing Medical University, Nanjing, Jiangsu 210000, China

## Abstract

Previous studies suggest that metformin may exert a protective effect on cisplatin-induced cytotoxicity in cancer cells, and this finding has led to a caution for considering metformin use in the treatment of cancer patients. However, in this paper we provide the first demonstration that metformin synergistically augments cisplatin cytotoxicity in the esophageal squamous cancer cell line, ECA109, under glucose-deprivation conditions, which may be more representative of the microenvironment within solid tumors; this effect is very different from the previously reported cytoprotective effect of metformin against cisplatin in commonly used high glucose incubation medium. The potential mechanisms underlying the synergistic effect of metformin on cisplatin-induced cytotoxicity under glucose-deprivation conditions may include enhancement of metformin-associated cytotoxicity, marked reduction in the cellular ATP levels, deregulation of the AKT and AMPK signaling pathways, and impaired DNA repair function.

## 1. Introduction

Esophageal cancer is a highly malignant and lethal disease, with an uneven worldwide distribution and particularly high incidence in China [1]. Although a significant improvement of treatment outcome has been reported due to the innovation of therapeutic modality over the last 3 decades, the 5-year overall survival remains dismal at 10% to 20% [2]. Therefore, there is a strong demand for new curative approaches for this disease.

Cisplatin (*cis*-dichlorodiammineplatinum, DDP) is the most commonly used chemotherapeutic agent in the treatment of a wide variety of solid tumors, including esophageal cancer. Cisplatin has multiple mechanisms of cytotoxicity, including generation of DNA and protein adducts and oxidative stress, which drive the activation of apoptosis cascades [3]. However, drug refractory is often observed in clinical settings, which may be associated with the heterogeneous microenvironment of solid tumors. Glucose-deprivation is a consequence of poor vasculature and limited glucose supply and is commonly observed in the solid tumor microenvironment.* In vivo*, normal serum glucose is usually maintained between 4 and 6 mM, whereas the glucose concentration within solid tumor has been observed to be markedly lower, typically lower than 0.5 mM [4–6]. These restricted glucose levels have been shown to induce cell cycle arrest, slow the cellular proliferation rate, and drive tumor cells into quiescence [7]. In addition, it has been well established that DDP, as a DNA-targeting agent, is more effective in rapidly dividing cells than in static cells and that the quiescent cells present in the low-glucose microenvironment within solid tumor are more resistant to DDP [7, 8] and may potentially be responsible for treatment failure.

Metformin is the most widely used oral hypoglycemic agent in type 2 diabetes because of its favorable toxicity profile and extremely low cost. In addition, it recently gained increasing interest from the medical community due to its potential antitumor effects [9]. A growing body of data suggests that metformin may elicit antitumor effects alone [9, 10] or synergistically improve the response of radiotherapy or chemotherapy [9, 11, 12]. However, recent studies showed an unexpected antagonistic effect of metformin on the cytotoxicity of DDP* in vitro* [13, 14], suggesting that caution should be taken when considering metformin for the treatment of diabetic cancer patients receiving DDP or as a potential adjuvant in DDP-based chemotherapeutic regimens. Here, we provide the first demonstration that, in contrast to the* in vitro* results obtained under artificially high glucose concentrations (10 to 25 mM), the treatment of esophageal squamous cell carcinoma (ESCC) cells with metformin synergistically augments DPP-induced cytotoxicity under low-glucose conditions (0.5 mM), which more accurately reflect the lower glucose levels present within the solid tumor microenvironment* in vivo* [4–6]. Therefore, the application of metformin in the treatment of patients with esophageal squamous cell carcinoma (ESCC) warrants further investigation.

## 2. Materials and Methods

### 2.1. Cell Line and Cell Culture

The ESCC cell line ECA109 was purchased from the Type Culture Collection of the Chinese Academy of Sciences (Shanghai, China). The cells were maintained in RPMI 1640 medium (KeyGEN Biology, Nanjing, China) supplemented with 10% fetal bovine serum (FBS, Gibco, Auckland, New Zealand) at 37°C in humidified air containing 5% carbon dioxide. RPMI 1640 medium with varying concentrations of glucose was obtained by mixing complete RPMI 1640 media with glucose-free RPMI 1640 medium at the appropriate volume proportions.

### 2.2. Cell Viability Analysis

The cell viability was assayed using the CCK-8 kit (KeyGEN Biology, Nanjing, China) according to the manufacturer's instructions. ECA109 cells were plated in 96-well plate for 24 hours, and medium was then replaced with normal complete RPMI 1640 medium containing 11.1 mM glucose (high glucose medium) or RPMI 1640 medium with 0.5 mM glucose (glucose-deprivation medium). The indicated concentration of metformin was then added to both groups, and 1 *μ*M DDP was added to specific wells that contained media alone or supplemented with indicated concentration of metformin. The cells were incubated for 48 hours, and then 10 *μ*L of CCK-8 was added directly to the 96-well plate. After the plate was incubated for 3 hours, the absorbance of the plates at 450 nm was read using a microplate reader (Biotek, MQX200).

### 2.3. ATP Assay

The cells were seeded in 6-well plates and allowed to grow for 24 hours. The incubation medium was then replaced with either fresh high glucose medium or glucose-deprivation medium. Metformin (200 *μ*M) was then added to specific wells, and, 24 hours after treatment, equal numbers of cells from each treatment group were lysed with ice-cold lysis buffer. Ten microliters of lysate supernatant was used for the ATP assay following the manufacturer's protocol (ATP Determination Kit, Beyotime Biotechnology, Jiangsu, China). Briefly, 10 *μ*L of the sample lysate supernatant was added to the 100 *μ*L reaction solution, and the fluorescence was measured. The background fluorescence, which was measured for 100 *μ*L of the standard reaction solution, was subtracted from the fluorescence of the sample, and the results were plotted as fold changes compared with the control group.

### 2.4. Western Blot

ECA109 cells were treated with or without 200 *μ*M metformin in high glucose medium or glucose-deprivation medium and the protein expression and phosphorylation of AMPK and AKT were evaluated by western blotting. Briefly, ECA109 cells were seeded in 6-well plates and cultured to approximately 50% confluence. The medium in specific wells was replaced with fresh high glucose or glucose-deprivation medium supplemented with or without 200 *μ*M metformin. The cells were then incubated for an additional 24 hours. Following the indicated treatments, cell protein extracts were prepared, and western blots were performed using 80 *μ*g of protein extract as previously described.

### 2.5. Immunofluorescence Assay

ECA109 cells were plated onto 35 mm^2^ plastic dishes and cultured to approximately 50% confluence. The medium was then replaced with high glucose medium or glucose-deprivation medium. After incubation for 6 hours, 200 *μ*M metformin and 1 *μ*M DDP were simultaneously added to the two groups of dishes. Three hours later, the cells were collected, fixed in 4% paraformaldehyde solution (Sigma-Aldrich, St. Louis, MO, USA) for 10 min at room temperature, and permeabilized in 0.1% Triton X-100 (Sigma-Aldrich, St. Louis, MO, USA) for 5 min. The cells were then blocked with 5% bovine serum albumin (BSA, Beyotime Biotechnology, Jiangsu, China) for 3 h, incubated with a primary antibody against *γ*-H2AX (Ser139, Cell Signaling Technology, MA, USA) overnight at 4°C, rinsed three times with PBS for 5 min per wash, and incubated with an Alexa Fluor 488-conjugated secondary antibody (Cell Signaling Technology, MA, USA) for 1 hour. The cells were then exposed to 1 *μ*g/mL DAPI (4′,6-diamidino-2-phenylindole, KeyGEN Biology, Nanjing, China) for nuclear DNA staining, rinsed three times with PBS for 5 min per wash, and visualized using a confocal fluorescence microscope (Leica Microsystems, Germany).

### 2.6. Statistical Analysis

The numerical results are presented as the means ± SD. A *t*-test was performed to compare the means of different groups using Prism 5.0 software (GraphPad Prism). For all statistical analyses, a *p* value less than 0.05 was considered statistically significant.

## 3. Results

In glucose-deprivation medium, metformin showed significantly enhanced cytotoxicity and synergistically augmented the cytotoxicity of DDP.

**Figure 1:**
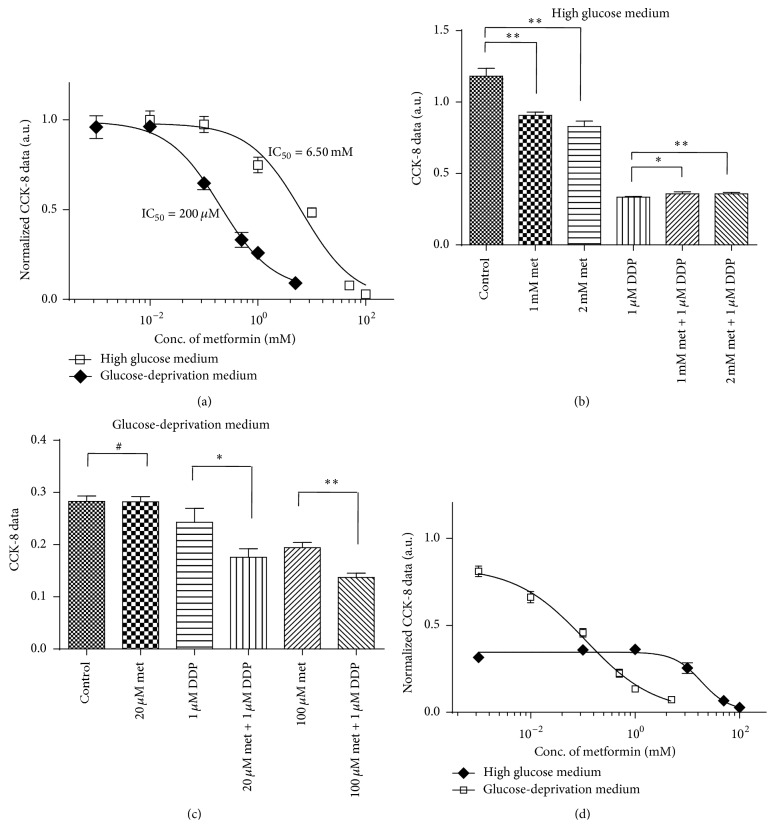
Metformin induced enhanced cytotoxicity in ECA109 cells and augments DDP-induced cytotoxicity in glucose-deprivation medium. (a) Dose-survival curves of ECA109 cells treated with varying concentrations of metformin in high glucose medium or glucose-deprivation medium; (b) treatment of ECA109 cells with 1 mM or 2 mM metformin led to a slight protective effect against DDP-induced cytotoxicity in high glucose medium; (c) treatment of ECA109 cells in glucose-deprivation medium with 20 *μ*M or 100 *μ*M metformin showed a synergistic cytotoxic effect with DDP. (d) Effect of various concentration of metformin on the cytotoxicity of 1 *μ*M DDP. The CCK-8 reading of each point was normalized to the reading of control cells maintained in high glucose and glucose-deprivation medium, respectively. # indicates no significant difference, *∗* indicates *p* < 0.05, *∗∗* indicates *p* < 0.01, met is short for metformin, and DDP is short for cisplatin.

First, we performed a dose-survival experiment with metformin on ECA109 cells under high glucose and low-glucose conditions. As shown in Figure 1(a), the dose-survival curve significantly shifted to the right with increases in the glucose level in the medium. The IC_50_ value of metformin in glucose-deprivation medium was approximately 200 *μ*M (95% CI: 150 to 267), whereas the IC_50_ increased to 6.21 mM (95% CI: 3.63 to 10.62) when medium was changed to normal RPMI 1640 medium with 11.1 mM glucose. Thus, in this study, we found that the* in vitro* cytotoxic effect of metformin on ECA109 increased approximately 32-fold in the glucose-deprivation medium compared with that observed in high glucose medium, as determined through the IC_50_ values.

Second, we found that, in high glucose medium, metformin slightly protected ECA109 cells against DDP cytotoxicity. As shown in Figure 1(b), 1 *μ*M DDP showed significant cytotoxicity, as determined by the cell viability, which was only 28.3% compared with the control cells. High concentrations of metformin (1 mM and 2 mM) led to moderate cytotoxicity on ECA109 cells because the observed cell viability was approximately 76.7% and 70.2% of that obtained for the control cells, respectively. However, we found that the combination of metformin and DDP did not induce synergistic cytotoxicity in ECA109 cells. In contrast, cotreatment with metformin appeared to slightly protect ECA109 cells from DDP-induced cytotoxicity, as demonstrated by the cell viability of the combination groups, which were 30.2% and 30.3% of that obtained for the control cells. This protective effect of metformin was slightly, albeit significantly, higher than that observed in the cells treated with 1 *μ*M DDP alone, as demonstrated by the *t*-test and *p* values, which reached 0.022 and 0.006, respectively.

Third, we found that the cytotoxicity effect of DDP was markedly lower in glucose-deprivation medium than in high glucose medium because the viability of the cells treated with 1 *μ*M DDP was approximately 70.1% of that obtained for the control cells, a value that is clearly higher than the viability of 28.3% obtained under high glucose conditions. These findings are consistent with previous reports that quiescent cells are more resistant to DDP [7, 8]. However, treatment with metformin was shown to synergistically promote DDP-induced cytotoxicity under glucose-deprivation conditions. As shown in Figure 1(c), the treatment of ECA109 cells with 20 *μ*M metformin did not promote overt cytotoxicity, whereas the addition of 1 *μ*M DDP with 20 *μ*M metformin led to a significant synergistic cytotoxic response. Similarly, a moderate level of cytotoxicity was observed in cells treated with 100 *μ*M metformin, while it was synergistically augmented when this treatment was combined with 1 *μ*M DDP, as demonstrated by the finding that the cytotoxicity of the combined treatment was significantly greater than the cytotoxicities observed with each individual treatment. The survival curves of ECA109 cells treated with 1 *μ*M DDP combining with various concentration of metformin were presented in Figure 1(d). As shown, under high glucose condition, increase of metformin concentration did not show any augmentation of the cytotoxicity of 1 *μ*M DDP until it reached high concentration to millimolar range. In contrast, metformin starts to show synergistic effect on the cytotoxicity of 1 *μ*M DDP from a concentration as low as 10 *μ*M under glucose-deprivation condition.

In glucose-deprivation medium, metformin caused a significant reduction of ATP synthesis and interfered with the AMPK and AKT signaling pathways in ECA109 cells.

**Figure 2:**
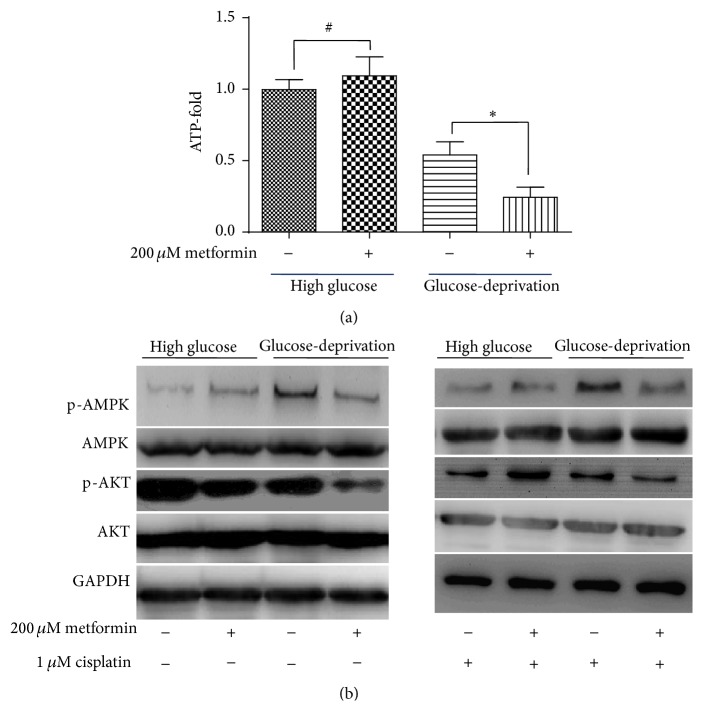
Metformin treatment (200 *μ*M) causes a significant reduction in the intracellular ATP levels and interferes with AMPK and AKT signaling. (a) Treatment of ECA109 cells with 200 *μ*M metformin showed no significant effect on ATP production in high glucose medium. Glucose-deprivation caused a reduction in the ATP levels in ECA109 cells, and the addition of metformin in glucose-deprivation medium caused a marked reduction in the ATP levels in ECA109 cells. (b) Activation status of AMPK and AKT in ECA109 cells treated with or without metformin (200 *μ*M) and cisplatin (1 *μ*M) under both high glucose and glucose-deprivation conditions. # indicates no significant difference; *∗* indicates *p* < 0.05.

The cellular ATP levels of ECA109 cells were measured after treatment with 200 *μ*M metformin for 24 hours in either high glucose medium or glucose-deprivation medium. As shown in Figure 2(a), 200 *μ*M metformin treatment did not induce a significant change in the cellular ATP level in ECA109 cells cultured in high glucose medium. In contrast, a significant reduction in the ATP levels was observed in ECA109 cells treated with 200 *μ*M metformin in glucose-deprivation medium.

To gain insight into the molecular mechanisms that underlie the differential effects of metformin on DDP-induced cytotoxicity in varying levels of glucose, we studied the activation status of AMPK and AKT kinases, which play a well established role in energy sensing and survival signaling and are involved in cell proliferation and cellular resistance to DDP. As shown in Figure 2(b), the addition of 200 *μ*M metformin to high glucose medium further augmented AMPK activation, whereas the addition of 200 *μ*M metformin under glucose-deprivation conditions led to a significant inhibition of AMPK activation. As for AKT, the addition of 200 *μ*M metformin slightly inhibited AKT phosphorylation in high glucose medium, whereas the addition of 200 *μ*M metformin under glucose-deprivation conditions led to a significant inhibition of AKT phosphorylation. Addition of 1 *μ*M DDP significantly altered AKT activation status. AKT phosphorylation was significantly inhibited by DDP addition under high glucose condition. It was coincident with our observation that DDP showed most significant cytotoxicity on cells in high glucose medium. We also found that 200 *μ*M metformin addition in the high glucose medium maintained the phosphorylation of AKT from the inhibition effect of DDP. This may be due to the intracellular reductive state caused by metformin [13], which elicits the protective effect against the toxicity effect of DDP. As expected, AKT phosphorylation level was lowest in the metformin and DDP combination group under glucose-deprivation condition, which was coincident with the observation that the combination group showed significant synergistic effect on cytotoxicity.

Under glucose-deprivation conditions, metformin significantly impaired the DDP-induced DNA damage repair process.

**Figure 3:**
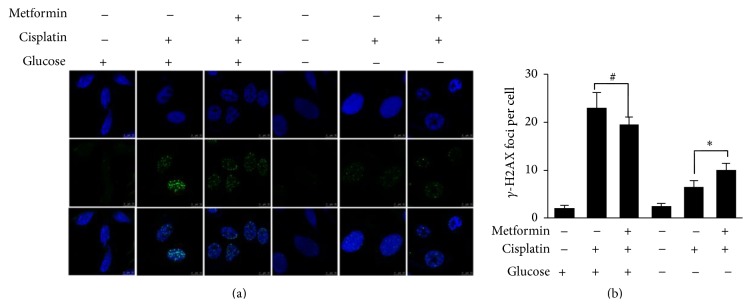
Metformin significantly retards the DNA repair process in glucose-deprivation medium. In high glucose medium, 200 *μ*M metformin addition slightly reduced the expression of *γ*-H2AX foci caused by 1 *μ*M DDP, while, under glucose-deprivation condition, addition of 200 *μ*M metformin significantly elevates the expression of *γ*-H2AX foci formation caused by 1 *μ*M DDP. *∗* indicates *p* < 0.05; # indicates no significant difference found.

DDP induces DNA cross-links and does not directly induce DNA double-strand breaks (DSB) or phosphorylation of H2AX. H2AX phosphorylation within cells treated by DDP has been considered to be associated with active DNA repair process, which includes nucleotide excision repair (NER) and nonhomologous end joining (NHEJ) [15]. Therefore, the extent of *γ*-H2AX was a surrogate marker to assess the DNA damage in DDP-treated cells. We examined the extent of *γ*-H2AX in control cells or cells treated for 3 hours with 200 *μ*M metformin with or without 1 *μ*M DDP under both high glucose and glucose-deprivation conditions. As shown in Figure 3, our experiments showed that there were nearly no *γ*-H2AX foci within intact ECA109 cells maintained in both high glucose and glucose-deprivation medium. We observed the expression of *γ*-H2AX foci was significantly enhanced in cells treated with DDP under high glucose condition; addition of 200 *μ*M metformin was observed to slightly reduce the expression of *γ*-H2AX foci (*p* = 0.074). Under glucose-deprivation condition, addition of DDP significantly enhanced the expression of *γ*-H2AX foci. However, in contrast to the slight protective effect of metformin observed under high glucose condition, 200 *μ*M metformin addition significantly elevates the number of *γ*-H2AX foci caused by 1 *μ*M DDP under glucose-deprivation condition (*p* < 0.05). Collectively, we observed that, under high glucose condition, 200 *μ*M metformin addition slightly reduced the expression of *γ*-H2AX foci caused by 1 *μ*M DDP, while, under glucose-deprivation condition, addition of 200 *μ*M metformin significantly enhanced the expression of 1 *μ*M DDP that caused *γ*-H2AX foci formation.

## 4. Discussion

Dozens of studies depict the antitumor effects of metformin and the potential underlying mechanisms. However, most of these studies were performed in common cell culture medium, which contains an abundance of glucose (10 to 25 mM). This* in vitro* cell culture condition with an artificially high glucose level may mask many physiological biochemical reactions that occur* in vivo*, particularly those associated with energy metabolism. The results from these studies showed that metformin exerts antitumor effects at high drug concentrations in the millimolar range, which are not physiologically achievable in the plasma of diabetes patients who use metformin (micromolar concentrations) [16]. Additionally, a series of studies warned that metformin may exert a cytoprotective effect against the common chemotherapy agent, DDP [13, 14, 17]. Based on these findings, some researchers have criticized the use of metformin in future anticancer therapy investigation. However, in this study, we showed that metformin-induced cytotoxicity is enhanced under glucose-deprivation conditions. Based on the calculated IC_50_ values, the cytotoxic effect of metformin on ECA109 cells increased approximately 32-fold in glucose-deprivation medium compared with that obtained for cells cultured in high glucose medium. Additionally, we showed that metformin synergistically augments DPP-induced cytotoxicity under glucose-deprivation conditions.

Although the precise mechanism underlying the antitumor effects of metformin remains elusive, convincing data suggest that the modulation of energy metabolism may play an important role in metformin's pharmaceutical effect [16]. Previous works suggested that metformin exerts its biochemical effect in cancer cells primarily via inhibition of respiratory-chain complex 1 in a dose-dependent manner, thus reducing mitochondrial oxidative phosphorylation (OXPHOS) and ATP production [16]. Under high glucose conditions, cancer cells can elevate their glycolytic efficacy via activation of AMPK signaling to maintain ATP production and achieve energy homeostasis [18, 19]. However, under glucose-deprivation conditions fuel supplies may be more tightly restricted and promoting glycolysis to meet ATP production demands may be unsustainable and lead to energy collapse and cell death [20]. Indeed, our results support this hypothesis, and we observed a marked reduction in ATP levels after 24 hours of treatment with 200 *μ*M metformin in glucose-deprivation medium.

Consistent with previous works [13, 14], our data suggest that metformin exerts a slightly cytoprotective effect against DDP in ECA109 cells cultured under high glucose conditions. Of note, this cytoprotective effect was observed at high drug concentrations of metformin in the millimolar range. Damellin et al. [13] suggested that the major mechanism underlying metformin-induced DDP resistance results from a significant increase in glycolysis and the intracellular NAD(P)H levels with a concomitant increase in the levels of reduced intracellular thiols, leading to decreased DDP-DNA adduct formation. Alternatively, work by Janjetovic et al. [14] suggests that metformin reduces DDP-induced anticancer activity* in vitro* through the AMPK-independent upregulation of the AKT survival pathway, because metformin induces AKT activation in DDP-treated cells and treatment with an AKT inhibitor abolishes the antioxidant and antiapoptotic effects of metformin. In this study, we found that treatment with 200 *μ*M metformin induced slight inhibition of the phosphorylation of AKT and slightly enhanced the activation of AMPK in high glucose medium. However, under glucose-deprivation conditions, the addition of metformin caused cellular energy homeostasis collapsing and induced significant synergistic cytotoxicity, and a significant inhibition of both AMPK and AKT phosphorylation was observed.

Convincing evidence indicates that the activation status of AKT is tightly associated with cellular DNA DSB repair function in cells [21]. To evaluate the effect of metformin on DNA repair function under different glucose conditions, we examined the formation of *γ*-H2AX foci in ECA109 cells that were treated for 3 hours with DDP in combination with metformin under both glucose conditions. We found a significant reduction in the formation of *γ*-H2AX foci, which serve as DSB repair cassettes, in glucose-deprivation medium compared with that found in the high glucose medium. Thus, our results suggest that, under glucose-deprivation conditions, when glycolytic fuel was limited, the energy supply was significantly restricted in ECA109 cells treated with 200 *μ*M metformin, and phosphorylation of AKT was significantly inhibited and then the DNA repair process was severely impaired. In line with this, we observed a significant synergistic cytotoxic effect following combined treatment with metformin and DDP in glucose-deprivation medium.

Recently, metformin has received increasing interest from the medical community due to its distinct anticancer effects, which include as the modulation of cancer cell energy metabolism [20], targeted killing of cancer stem cells [22], and inhibition of inflammatory response-associated cell malignancy transformation [10]. Accumulating clinical evidence reinforces the promising anticancer effects of metformin in a variety of cancers, such as esophageal [23], breast [11], and prostate ones [24]. A very recent retrospective single-institutional cohort study conducted by Van De Voorde et al. [23] showed that, in patients of esophageal cancer, use of metformin was associated with significantly improved overall survival, significantly increased distant metastasis-free survival, and a higher complete response rate.

To the best of our knowledge, this study provides the first demonstration of the varying effects of metformin on DPP-induced cytotoxicity against the ESCC cell line ECA109, under different glucose levels. In contrast to the cytoprotective effect of metformin observed under high glucose conditions, we showed that metformin synergistically augments the cytotoxicity of DDP in cells cultured under glucose-deprivation conditions. A possible mechanism underlying this difference may be associated with a reduction of ATP production, the inhibition of AKT activation, and the impediment of DNA repair processes. Thus, the use of metformin as a new therapeutic or adjuvant modality in esophageal cancer treatment warrants further investigation.

## References

[1] Jemal A., Center M. M., DeSantis C., Ward E. M. (2010). Global patterns of cancer incidence and mortality rates and trends. *Cancer Epidemiology Biomarkers & Prevention*.

[2] Jemal A., Siegel R., Xu J., Ward E. (2010). Cancer statistics, 2010. *CA Cancer Journal for Clinicians*.

[3] Siddik Z. H. (2003). Cisplatin: mode of cytotoxic action and molecular basis of resistance. *Oncogene*.

[4] Burgess E. A., Sylven B. (1962). Glucose, lactate, and lactic dehydrogenase activity in normal interstitial fluid and that of solid mouse tumors.. *Cancer Research*.

[5] Hirayama A., Kami K., Sugimoto M. (2009). Quantitative metabolome profiling of colon and stomach cancer microenvironment by capillary electrophoresis time-of-flight mass spectrometry. *Cancer Research*.

[6] Schroeder T., Yuan H., Viglianti B. L. (2005). Spatial heterogeneity and oxygen dependence of glucose consumption in R3230Ac and fibrosarcomas of the Fischer 344 rat. *Cancer Research*.

[7] Trédan O., Galmarini C. M., Patel K., Tannock I. F. (2007). Drug resistance and the solid tumor microenvironment. *Journal of the National Cancer Institute*.

[8] Tannock I. (1978). Cell kinetics and chemotherapy: a critical review. *Cancer Treatment Reports*.

[9] Kourelis T. V., Siegel R. D. (2012). Metformin and cancer: new applications for an old drug. *Medical Oncology*.

[10] Hirsch H. A., Iliopoulos D., Struhl K. (2013). Metformin inhibits the inflammatory response associated with cellular transformation and cancer stem cell growth. *Proceedings of the National Academy of Sciences of the United States of America*.

[11] Jiralerspong S., Palla S. L., Giordano S. H. (2009). Metformin and pathologic complete responses to neoadjuvant chemotherapy in diabetic patients with breast cancer. *Journal of Clinical Oncology*.

[12] Teixeira S. F., Guimarães I. D. S., Madeira K. P., Daltoé R. D., Silva I. V., Rangel L. B. A. (2013). Metformin synergistically enhances antiproliferative effects of cisplatin and etoposide in NCI-H460 human lung cancer cells. *Jornal Brasileiro de Pneumologia*.

[13] Damelin L. H., Jivan R., Veale R. B., Rousseau A. L., Mavri-Damelin D. (2014). Metformin induces an intracellular reductive state that protects oesophageal squamous cell carcinoma cells against cisplatin but not copper-bis(thiosemicarbazones). *BMC Cancer*.

[14] Janjetovic K., Vucicevic L., Misirkic M. (2011). Metformin reduces cisplatin-mediated apoptotic death of cancer cells through AMPK-independent activation of Akt. *European Journal of Pharmacology*.

[15] Huang X., Okafuji M., Traganos F., Luther E., Holden E., Darzynkiewicz Z. (2004). Assessment of histone H2AX phosphorylation induced by DNA topoisomerase I and II inhibitors topotecan and mitoxantrone and by the DNA cross-linking agent cisplatin. *Cytometry A*.

[16] Foretz M., Guigas B., Bertrand L., Pollak M., Viollet B. (2014). Metformin: from mechanisms of action to therapies. *Cell Metabolism*.

[17] Lesan V., Ghaffari S. H., Salaramoli J. (2014). Evaluation of antagonistic effects of metformin with Cisplatin in gastric cancer cells. *International Journal of Hematology-Oncology and Stem Cell Research*.

[18] Sahra I. B., Marchand-Brustel Y. L., Tanti J.-F., Bost F. (2010). Metformin in cancer therapy: a new perspective for an old antidiabetic drug?. *Molecular Cancer Therapeutics*.

[19] Dykens J. A., Jamieson J., Marroquin L., Nadanaciva S., Billis P. A., Will Y. (2008). Biguanide-induced mitochondrial dysfunction yields increased lactate production and cytotoxicity of aerobically-poised HepG2 cells and human hepatocytes in vitro. *Toxicology and Applied Pharmacology*.

[20] Birsoy K., Possemato R., Lorbeer F. K. (2014). Metabolic determinants of cancer cell sensitivity to glucose limitation and biguanides. *Nature*.

[21] Toulany M., Rodemann H. P. (2013). Potential of Akt mediated DNA repair in radioresistance of solid tumors overexpressing erbB-PI3K-Akt pathway. *Translational Cancer Research*.

[22] Song C. W., Lee H., Dings R. P. M. (2012). Metformin kills and radiosensitizes cancer cells and preferentially kills cancer stem cells. *Scientific Reports*.

[23] Van De Voorde L., Janssen L., Larue R. (2015). Can metformin improve ‘the tomorrow’ of patients treated for oesophageal cancer?. *European Journal of Surgical Oncology*.

[24] Kaushik D., Karnes R. J., Eisenberg M. S., Rangel L. J., Carlson R. E., Bergstralh E. J. (2014). Effect of metformin on prostate cancer outcomes after radical prostatectomy. *Urologic Oncology*.

